# Nanotechnology for the treatment of melanoma skin cancer

**DOI:** 10.1007/s40204-017-0064-z

**Published:** 2017-03-16

**Authors:** Lucas B. Naves, Chetna Dhand, Jayarama Reddy Venugopal, Lakshminarayanan Rajamani, Seeram Ramakrishna, Luis Almeida

**Affiliations:** 1 CAPES Foundation, Ministry of Education of Brazil, Brasília, Brazil; 2 Centre for Textile Science and Technology, University of Minho, Braga, Portugal; 3 Department of Mechanical Engineering, Center for Nanofibers & Nanotechnology, National University of Singapore, Singapore, 117576 Singapore; 4 Anti-Infectives Research Group, Singapore Eye Research Institute, Singapore, 169856 Singapore; 5 Guangdong-Hongkong-Macau Institute of CNS Regeneration (GHMICR), Jinan University, Guangzhou, 510632 China

**Keywords:** Chemotherapy, Drug delivery systems, Kinase inhibitors, Skin cancer therapy, Topical delivery

## Abstract

Melanoma is the most aggressive type of skin cancer and has very high rates of mortality. An early stage melanoma can be surgically removed, with a survival rate of 99%. This literature review intends to elucidate the possibilities to treat melanoma skin cancer using hybrid nanofibers developed by advanced electrospinning process. In this review we have shown that the enhanced permeability and retention is the basis for using nanotechnology, aiming topical drug delivery. The importance of the detection of skin cancer in the early stages is directly related to non-metastatic effects and survival rates of melanoma cells. Inhibitors of protein kinase are already available in the market for melanoma treatment and are approved by the FDA; these agents are cobimetinib, dabrafenib, ipilimumab, nivolumab, trametinib, and vemurafenib. We also report a case study involving two different approaches for targeting melanoma skin cancer therapy, namely, magnetic-based core–shell particles and electrospun mats.

## Introduction

Melanoma, originated from the malignant transformation of melanocytes, is one of the most aggressive skin cancers, notorious for its high multidrug resistance (MDR), easy to relapse and low survival rate. Nearly 76,100 newly diagnosed cases of melanoma were reported in the United States in 2014 with an estimated 9710 expected deaths. The trend worldwide is to use more and more nanotechnologies for several biomedical applications. Nanofibers are ideal for this purpose because their dimensions are similar to the components of native extracellular matrix and mimic its fibrillar structure, providing essential cues for cellular organization and survival function. Nanotechnology is a field of applied science focused on the design, synthesis, developing, fabrication, characterization, and application of materials and devices on the nanoscale. It has the potential to make a significant impact on healthcare by delivering changes in disease diagnosis and monitoring, implants and regenerative medicine, drug delivery, as well as research tools for drug discovery and biomedical science. In the healthcare environment, the use of nanotechnology can create new alternative treatments, which can be more efficient, minimizing the side effects and reducing the treatment cost significantly either to the government or the patients. Increasingly scientists have focused on developing new drugs and new ways to release them to patients in a less invasive route, with the use of nanotechnology; such possibility is increasing day by day in healthcare. Nanotechnology has been applied to most conventional therapies for melanoma. It has been demonstrated that the nanodelivery of drugs for chemotherapy, targeted therapy, immunotherapy, and photodynamic therapy has greatly increased treatment efficacy. Targeting in nanotechnology refers to the spatial localization of the nanoparticles within the intentional sites and is distinct from molecularly targeted drugs. Targeted drug means blocking essential biochemical pathways or mutant proteins that are required for tumor cell growth. The review summarizes the types of skin cancer and controlled delivery of drug-loaded nanofibers for melanoma skin cancer therapy (Naves and Almeida 2015).

## The skin

Skin, the largest organ in the body, has a surface area of about 1.8 m^2^ and occupies 8% of the total body mass of an adult. The functions are foremost as a barrier, preventing pathogens from entering the body and also a sensory organ and a regulator for water retention and heat loss. Skin is an attractive model organ to test novel concepts of regenerative medicine, with a particular emphasis on skin tissue regeneration for acute or chronic wounds. Chronic wounds present a worldwide growing health and economic problem because of a steadily increasing number of patients, high morbidity and risk of amputations, unsatisfactory results of existing therapies, and heavy socioeconomic burden. The potential route for drug delivery through the skin surface allows systemic and local drug delivery using nanoparticles, which can be rapidly reached into opened follicles of the hair and skin diseases. Nanoparticles higher than 10 nm are difficult to penetrate the skin barrier, the stratum corneum (SC), however, by applying localized massage, it is possible to permit these nanoparticles localized into hair follicle openings to play as nanoparticle reservoir. One of the primary and most important functions of skin in a mammalian is to provide protective barrier against fungi, bacteria, UV radiation, and nanoparticles coming from the natural environment.

In human’s skin, the hair follicles cover only 0.1% of the skin surface, however, target drug delivery can be performed through follicular route, to achieve a better drug penetration. By pulling out the hair sheath, it is possible to make a significant penetration of medicines in a size smaller than 40 nm into epidermal cell, to reach, and facilitate a higher topical nanoparticle delivery through skin. Mechanical stress on the skin is necessary, which can be achieved by massage, skin flexing, or follicles pulled out. Over the last few years, the interest in a target by follicular delivery has increased as it might be a potential delivery route into sebum to control the skin diseases (Naves and Almeida 2015).

The surface of the skin has its pH influenced by the sweat, hydration, gender, and anatomical site (Tinkle et al. 2003). The pH of the skin is acidic, around 4.2–5.6 (Schmid-Wendtner and Korting 2006). The pH of the skin supports penetration of nanoparticles and by a solution of lowered pH, it is possible to achieve a decreased electrostatic force (Murphy et al. 2010). The skin structure is divided into two main layers, the epidermis and the dermis. The epidermis is separated from the dermis by a basement membrane, surrounded by extracellular lipid matrix, containing SC cells, keratinocytes, and corneocytes (arranged in bilayers, packed into the extracellular lipid matrix (Elias and Menon 1991), and in some studies this is reported as “brick and mortar” arrangement). The dermis is located under the epidermis, which is formed by a variety of connective tissues, as the lymphatic system, nerves, blood vessels, and many types of cells.

The topical drug delivery has been practiced since the end of 70´s, using transdermal patches, and nanoparticles have been developed in the last decades to administer readily available local therapies (Roy et al. 1996). Desquamation of the skin is a natural process that happens in humans, in the SC layers. The renewal process is complete by approximately 14 days; it is important to note that this period is age and anatomical dependent (Reddy et al. 2000). Through this natural process, the corneocytes can provide elimination of certain matters such as cancer cells, solid particles, and pathogens.

## Melanoma skin cancer

Skin cancers are by far the most common malignancy of humans, particularly in the white population, with over a million cases detected each year. Skin cancers are named according to the cell from which they arise and the clinical behavior. The three commonest types are basal cell carcinomas (BCCs) and squamous cell carcinomas (SCCs) (both referred as non-melanocytic skin cancer—NMSC) and cutaneous malignant melanomas (CMs) (also referred to malignant melanoma of the skin or melanoma). Treating melanoma skin cancer is possible due to the application of emerging nanotechnologies, which can enhance cancer cell uptake, minimize toxicity, increase the circulation tissue, etc. (Tran et al. 2009). The melanoma cancer is formed either by dysfunction of dysplastic nevi or a single melanocyte. The melanocytes, located at the base of the epidermis, are responsible for producing the melanin pigment that can be found in our hair, eyes, and skin (Eggermont et al. 2014). The vascularization of the tumor happens naturally, by passive diffusion, where tumor cells obtain all the requisite nutrients to growth till it reaches the size range 2 mm^3^(Jones and Harris 1998). When the tumor cells are bigger than 2 mm^3^, the next happening is angiogenesis, which is new blood vessel formation, to supply enough nutrients to the cancer cells, increasing the mass of the tumor once these areas become richly vascularized ( Maeda et al. 2000).

**Fig. 1:**
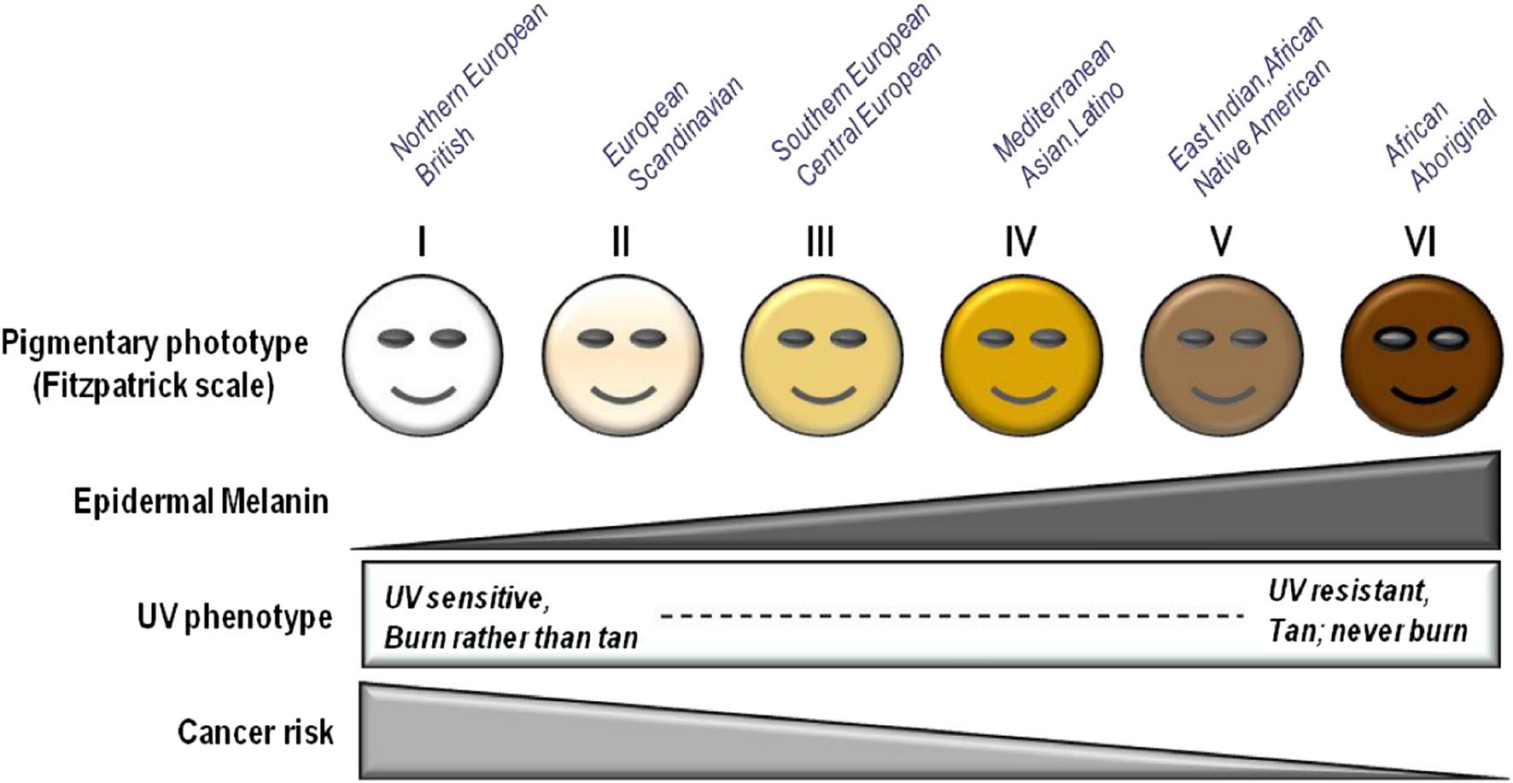
Correlation between incidence of UV rays, skin color, and skin cancer risk [Image open access: (D’Orazio et al. 2013)]

In Fig. 1 it is possible to observe the influence of skin pigmentation and its correlation with skin cancer risk. Individuals having low levels of melanin in the dermis, namely fair-skinned people, tend to skin-burn rather than become tan, after UV exposure. Data suggested that mutations that contribute to tanning impairment and to fair complexion, may be attributed to less efficient DNA repair in melanocytes. Individuals with melanocortin 1 receptor (MC1R) have less ability to block UV photons, therefore, they suffer higher realized doses of UV radiation, also accumulating more mutations from UV exposure due to defective DNA repair (D’Orazio et al. 2013).

Among all types of skin cancer, melanoma is the type of cancer with a higher rate of metastatic effect. Once located in the dermis, it has the potential to spread through the other body sites by entering the lymphatic system and bloodstream, for this reason, it is important to be painstaking to the Breslow’s tumor thickness which can be classified according to Table 1 (Garbe and Eigentler 2007); using the Breslow’s tumor thickness it is possible to make a prognosis of the patients, if there is a possibility of metastasis by knowing the survival rate for patients diagnosed with melanoma skin cancer. Surgery offers a good chance of recovery at the early stage of melanoma. However, for advanced metastatic melanoma, only modest results are obtained with DTIC (1975), recombinant interferon α-2b (1995), and high-dose interleukin 2 (HD IL-2) (1998), the only three conventional therapeutic agents approved by the Food and Drug Administration (FDA) for metastatic melanoma.

**Table 1:** Breslow’s tumor thickness

Stage	Depth
I	Tumor with ≤1.0 mm
IIA	1.01–2.0 mm
IIB	2.01–4.0 mm
III	4.0 mm ≤ tumor depth

Photo-aging is a process that happens to us, due to inflammation and reactive oxygen species. The photo-aging may provoke metabolic changes, DNA, and collagen damage. It is often associated with skin cancer (Green 1991). For a better understanding of cancer cell proliferation, it is important to understand the meaning of BRAF and protein kinase. In humans, BRAF is a gene found in cancer cells, it is critical, which is responsible for making a protein called BRaf, which is involved in the cells of human body, sending signals to activate the cell proliferation. Another factor that might influence the proliferation of cancer cells is the protein kinase, which is a group of enzymes, responsible for the phosphorylation process, consisting in the transformation of the phosphate group onto proteins. This process is responsible for many important cellular functions, such as cycle progression, metabolism, differentiation, and apoptosis.

## Targeting strategies

The tumor can be targeted via active or passive strategies to ensure that (1) the anticancer drugs provoke apoptosis only to tumor cells without damaging the functions of the surrounding cells; (2) the drugs are delivered to the tumor cells without overdose concentrations and minimize the loss of activity (Moghimi et al. 2001). The principle of active and passive targeting can be seen in Fig. 3.

### Active targeting

Active targeting nanotechnology involves conjugation of ligands such as peptides, antibodies, sugars, aptamers, or other small molecules to nanoparticles allowing the homing of the drug to the target site (Ojea-Jimenez et al. 2013). An ideal ligand–receptor should be selected to target the nanoparticles only to the malignant cells, avoiding the health cells. Some antibodies that we can mention for targeting melanoma cells are trastuzumab, rituximab, and bevacizumab, which are all approved by the FDA. Although antibodies have been used to direct the carriers in a site-specific manner, there are some limitations such as the high cost, the challenges of production in large scale, as well as the complex and large structure of monoclonal antibodies (Li et al. 2010). Particularly in the case of melanoma, other ligands can be harnessed for active targeting. Over the last few years, a significant progress has been done regarding the active targeting field, however, though it is still needed to specific molecular target expressed by melanoma cells. This can be the breaking point to the development of more efficient drug delivery system (DDS).

### Passive targeting

**Fig. 2:**
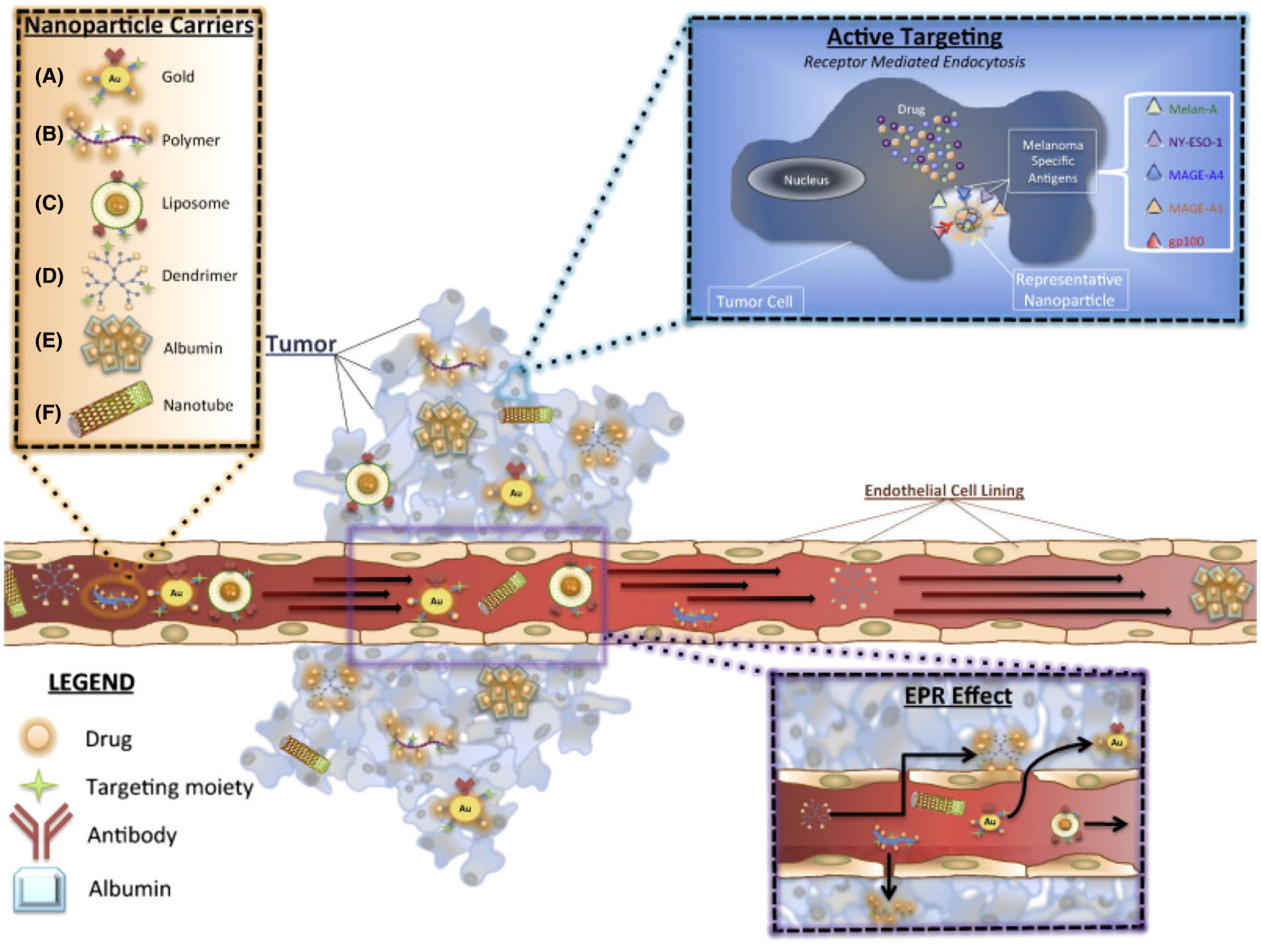
Drug delivery is increased by active and passive targeting to tumor site. Active targeting is achieved using known melanoma antigens, resulting in receptor-mediated endocytosis into the tumor cells. In passive targeting, the EPR effect is responsible for a significant accumulation of the drug at the tumor site [with Permission from Elsevier (Brys et al. 2016)]

Passive targeting is possible to achieve with the use of nanoparticles as a natural result of their biochemical properties in combination with EPR effect (Maeda 2001), shown in Fig. 2. The EPR effect is due to the leaky vasculature found within tumors where gaps between the endothelial cells may be 800 nm. As a result, there is a defective vascularized system, having a poor lymphatic drainage, thereafter, the nanoparticles enter into extracellular space of the tumors and remain within the tumor site, opposite to the normal tissue where the nanoparticles would remain in the vasculature. Doxorubicin (Doxil ^®^) is an anticancer drug that can be exploited for melanoma treatment and it is approved by the FDA. Clinically, it was shown that passive targeting of PEGylated liposomal doxorubicin has toxicity profile over standard doxorubicin (Brys et al. 2016).

### Drugs used for chemotherapy treatment

The chemotherapy treatment consists of slowing down or stopping the growth of cancer cells which can divide quickly. These chemicals have as side effect the damage of healthy cells. Targeting in nanotechnology refers to the spatial localization of the NP within the intentional sites and is distinct from molecularly targeted drugs. Targeted drug means blocking essential biochemical pathways or mutant proteins that are required for tumor cell growth. The aforementioned vemurafenib and dabrafenib are targeted drugs for melanoma patients with mutant BRAFV600E. The side effects related to the chemotherapy treatment often occur when the chemo is over. The advantages of this process are better shown in Table 2 (National Cancer Institute 2007).

**Table 2:** Types of chemotherapy which are applied to control skin cancer

Type of chemo	Why is it used?
Control cancer	It is used for cancer treatment and does not spread to other parts of the body, as it occurs in metastatic patients, slowing down the process or destroying the malignant cells that have been already scattered to the other sites inside the body
Cure cancer	When the drugs are delivered by chemotherapy they completely destroy the cancer cells and they can no longer be detected inside the patient’s body
Palliative care	Often used to shrink tumors when they are provoking pain to the patient, and the cure is not possible to be achieved

The properties of some drugs are available in the market for chemotherapy treatment; these drugs are approved by the FDA (Food and Drug Administration) in the United States; they are Cobimetinib, Ipilimumab, Nivolumab, Trametinib, and Vemurafenib etc. (Hanson et al. 2002).

#### Cobimetinib

Cobimetinib inhibits the growth and decrease the tumor cell proliferation; this chemical is a small molecule, which can be taken orally, usually taken once a day for the first 21 days of a treatment cycle of 28 days, so it should be suspended for 1 week in raw. The physicians recommend to take it always at the same time on every daily treatment. It is a bioactive inhibitor, poses a potential antineoplastic activity, classified as kinase inhibitors. By inhibiting the mitogen-activated protein kinase, it binds and inhibits the MEK 1 catalytic activity, resulting in a phosphorylation, which basically consists of a post-translational modification, turning many protein enzymes off and on; by doing so, it is possible to alter their activity and function. Many studies have shown that using this agent, it is possible to achieve a better inhibition of BRAF mutation, often associated with many types of tumor types. In the United States, this medicine is named Cottelic. It is used for patients who have been diagnosed with melanoma cancer, is an effective treatment when the tumor cannot be removed, and for some metastatic patients. The effectiveness of the treatment can be reached due to its property of blocking the action of abnormal proteins that often send signals to cancer cells to reproduce in numbers. It helps to slow down or stop spreading the tumor cells through the other parts of the patient’s body (Haley and Frenkel 2008) to control melanoma cancer.

#### Dabrafenib

Dabrafenib is given to the patient twice a day around the same time. It is usually given through the mouth. It inhibits the BRAF activity, resulting in a decreased proliferation of those cells having a mutated BRAF gene. It is an important regulator of ERKs/MAP kinase, signaling its pathway. This medicine is also used for those patients who either cannot undergo surgical removal or the tumor cells have already been spreading to other body sites. It also helps in slowing down the proliferation and/or minimizing the tumor cells spreading under anticancer therapy (Health, n.d.-b).

#### Ipilimumab

Ipilimumab binds the T-lymphocyte-associated antigen 4 (CTLA4), important in the downregulation of the immune system to be expressed in T-cells, leading to T-lymphocyte (CTL) cytotoxic influencing the immune response, fighting the cancer cells. In the United States, this medicine is also known as Yervoy, having the abbreviation of MDX-CTLA-4. This treatment is given to the patient through injection pathway, however, severe side effects may occur. The patient must be aware and report immediately to their physicians of any abnormal and unexpected side effect. It is important to treat and prevent the side effects before they lead to life-threatening. This drug is released to the patients via the intravenous route. Most of the times, at maximum, four doses are given, every 3 weeks, where each procedure might take around one and half hour. This agent is classified by the FDA as monoclonal antibodies (moAb or mAb), once they form a clone of a unique nearby cell and are made by identical immune cells (Jain 2013).

#### Nivolumab

Nivolumab possesses antineoplastic activities and immune checkpoint inhibitors. It acts directly on the cell surface receptors, in the negative immunoregulatory process, the activity role happens at the programmed cell death-1 (PCD-1 and/or PD1), which is over-expressed in many types of cancer cells. The advantage of blocking the PD1 is preventing the tumor cell evasion from the host immunity; in this manner regulating the T-cell activation. The T-cells are specific antigen receptors, essential for the response of the immune system. It is also commercially known as Opdivo injection or Opdivo. It is often used in combination of Ipilimumab, aiming the melanoma treatment. When this combination is indicated by the doctors, the procedure usually is done in four doses separately, once every 3 weeks. Afterwards, the patient should undergo the treatment once every 2 weeks depending on the doctor’s prescription. On the other hand, if the drug is used alone, generally it is given once every 2 weeks for as long as the drug is prescribed by the clinician. It plays a role in the immune system against cancer cells (Health, n.d.-d).

#### Trametinib

Trametinib is an anticancer agent that also has antineoplastic activity and is considered as a potential inhibitor of mitogen-activated protein kinase. The kinase process activation has an important role in signaling pathways and regulating cell growth. This activation could happen at ERK/RAS/RAF/MEK. This agent is also known as Mekinist and is classified as kinase inhibitors, blocking the activity of abnormal proteins that send signals for multiplying cancer cells in an uncontrolled way. Usually it is taken orally in tablet once a month, according to the doctor’s prescription to control the skin cancer (Health, n.d.-e).

#### Vemurafenib

Vemurafenib is taken as a tablet, per approximately 1 month twice a day. This drug developed by small molecules with a significant property of BRAF inhibition, resulting in a reduction of cancer cell proliferation. This agent is also named as Zelboraf or kinase inhibitor RO5185426. Scientists know that around 60% of the melanoma cells are directly related to the BRAF gene mutation. In Table 3 the delivery methods, the inhibitor process, and the duration of treatments, depending on the physician description, are compared.

**Table 3:** Drugs approved by FDA (Health, n.d.-a; n.d.-c; n.d.-d; n.d.-e; n.d.-f))

Drug	Delivery method	Inhibitor	Duration of treatment based on physician prescription
Cobimetinib	Orally	Inhibits the mitogen/activated protein kinase, stopping or decreasing the tumor cell proliferation	Taken once a day for the first 21 days of a treatment cycle of 28 days
Dabrafenib	Usually oral	Inhibits the B/RAF activity, decreasing the proliferation of the cells having a muted BRAF gene	Twice a day, around the same time
Ipilimumab	Injection	It binds the T/lymphocyte/associated antigen 4 (CTLA) on T-cells	Given at maximum 4 doses, every 3 weeks
Nivolumab	Injection	The activity role happens at the programmed death-1 (PCD-1 and/or PD1	Usually done in 4 doses separately, once every 3 weeks
Trametinib	Orally	Blocking the activity of abnormal proteins that send signals for multiplying cancer cell in an uncontrolled way	Once a month
Vemurafenib	Orally	BRAF inhibitor	Per approximately 1 month Twice a day

### The use of nanotechnology

Nanotechnology, being one of the most impending technology today, shows an extremely huge potential in the field of tissue engineering. Nanostructures are significant due to their inherent properties such as the large surface area to volume ratio and the engineered properties such as fiber diameter, porosity, stability, hydrophilicity, and permeability. Among the various nanomaterial synthesis procedures available, electrospinning serves to be a most promising technique for designing natural and synthetic polymer-based nanofibrous scaffolds to engineer artificial organs for tissue engineering and drug delivery applications. Researchers took efforts over the last decades, focusing their works to kill cancer cells through more specific targeting. Nanotechnology has been exploited for both imaging and drug delivery, aiming the diagnosis and treatment of melanoma cancer (Liu et al. 2007). Nanoparticles are useful to provide delivery functioning carriers for tumor vasculature. Thus, these particles are possible to bind the polymer into malignancy cell membrane, to nuclear or cytoplasmic receptor sites, enabling to reduce toxicity to the normal tissue, once it is possible to increase the drug concentration to the target cells (Haley and Frenkel 2008).

Enhanced permeability and retention (EPR) is the basis of nanotechnology for delivering drugs to the body sites. It allows molecules to enter the interstitial tumor space, by suppressing the lymphatic filtration making it possible to keep the molecules in the malignant cells. Different types of pore dimensions at the vascularity can interfere the EPR, as well as the tumor location, size, and type (Maeda 2012). Optimized drug delivery is enhanced by normalizing the tumor vasculature, lymphatic and extracellular matrix; this is achieved using small particles in ranging size of 20 nm (Jain 2013). New engineered biomaterials are important to be developed and researched by the scientific community, aiming to develop an advancement on topical drug delivery, revealing skin function, biological information, and drug kinetics (Prow et al. 2011).

In 1902, electrospinning was patented in the United States (Harbert and Morrison 1944; Ramakrishna et al. 2006). Figure 4 shows an example of electrospinning process. Unlike the traditional porous structure, when dealing with nanofibers, due to their dynamic system, it is possible to achieve a higher porosity. The porous structure of the fibers' mesh surface area can be increased using the electrospinning process, and having a higher surface area, the nanofibers can be applied for different purposes (Pike 1999). Among all the nanofiber application, we can cite the healthcare environment, where these nanofibers can be used for drug delivery, tissue repair, and also for tissue engineering. Almost any soluble polymer of high molecular weight can be used for electrospinning (Ramakrishna et al. 2006).

Electrospinning process is interesting for researchers worldwide, due to its versatile and simple method to obtain ultra-thin fibers. Basically, it is based on the application of high voltage, aiming to create an electrically charged jet, the polymeric solution, which is collected in a collector with an opposite polarity (Barnes et al. 2007). After the application of electrical field, the polymer is ejected from the Taylor cone, traveling through the atmosphere, at this moment the evaporation of the solution occurs, and as a result of opposite polarity, the collector receives the solid polymer fibers, by doing so, it is possible to create fibers with size ranging from nanometers to micrometers, having a high surface area, porosity, and volume ratio (Zamani et al. 2013). Some variables can be modified in the electrospinning process such as solution flow rate, the voltage applied, and also the distance between the Taylor cone and the collector. If is necessary physical and chemical properties changing of the surface of the polymer, it is possible to apply some surface modification methods such as oxidation, ion sputtering, and plasma. All these treatments, applied to the electrospun materials do not change the inherent bulk properties (Ravichandran et al. 2012). It is only possible to form non-woven fibers, since the process is very chaotic due to the repulsive charge in the electrospinning jet path (Pike 1999). Functionalized surface with a ligand can be applied to the electrospun nanofibers as affinity membrane.

Producing material with size ranging from 1 to 100 nm, nanotechnology basically consists in utilization or creation of materials at the level of molecules and atoms. The ratio of the polymer chain and its functional group can be increased, once the diameter of the nanofiber is decreased. Polymer nanofibers have several advantages for drug delivery. The nanofibers obtained by the electrospinning process can be applied for wound healing and to topically drug delivery to the skin (Zhang et al. 2005). It is possible to form high porosity fiber, using two different processes. The first is to blend two different polymers; one of them is removed by dissolution in a solvent after the fiber formation, so it is necessary to use polymers with distinguished characteristics regarding the solubility, where one polymer should be soluble and the other one should be insoluble. The other manner to obtain high porosity surface area profile is using a high volatile solvent, where the regions rich with this solvent during the electrospinning process will transform into pores (Bognitzki et al. 2001).

Zeng and co-workers (Zeng et al. 2003) have shown in their study that the quality of nanofibers such as surface uniformity and reduced diameter can be achieved when combining Paclitaxel and Rifampin into poly(*l*-lactic acid). Several components can be electrospun using the coaxial technique, which allows different capillary channels to integrate into a core–shell structure. In addition to that, it is possible to provide momentary protection for some bioactive substances. By choosing which particular electrospinning technique is used, it is possible to determine the nanofiber application in skin cancer therapy (Zhang et al. 2005).

#### Nanoparticles for skin delivering

**Fig. 3:**
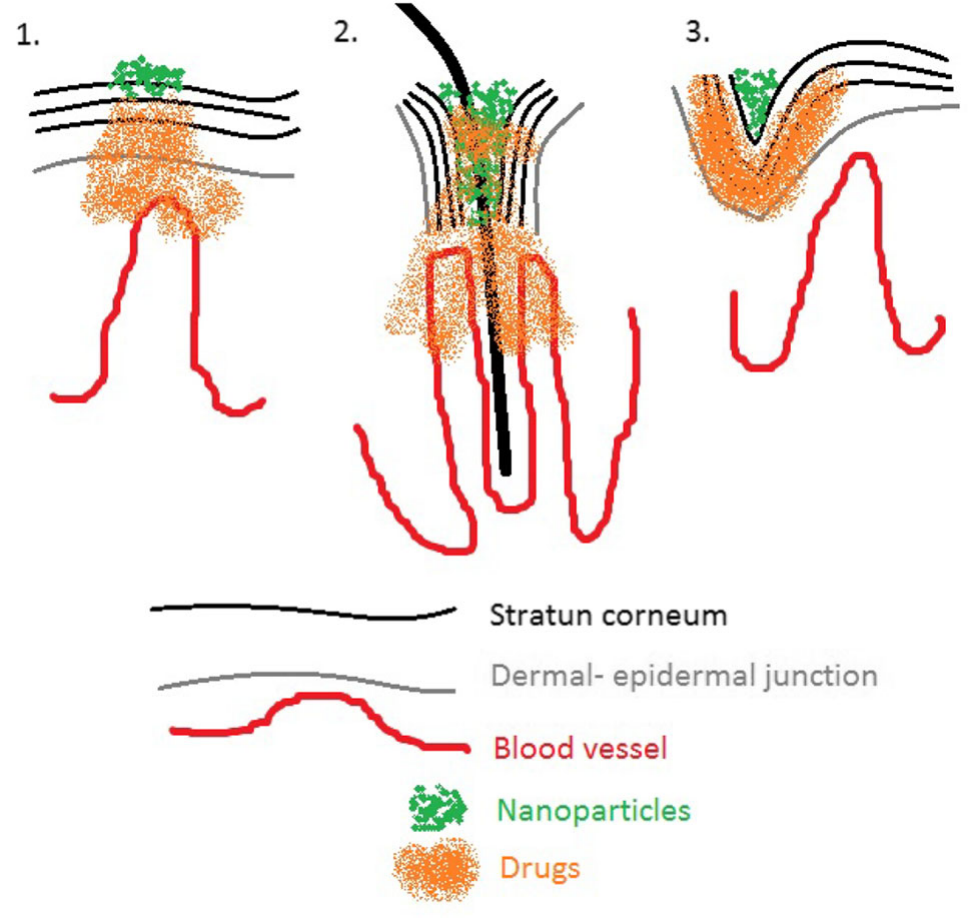
Topical nanoparticle drug delivery. SC surface (*panel 1*), hair follicles (*panel 2*), furrow delivery (*3*) [The *figure* shown here was adopted from Ref. Aboofazeli and Lawrence (1994)]

American National Standards Institute (ANSI) has defined nanoparticles, those particles which have an average size between 1 up to 100 nm (Rauscher and Roebben 2010). In order to delivery topical drugs, this procedure is possible to be achieved by three different sites, such as hair follicles opened, stratum corneum surface (SC), and last but not least by the furrows Aboofazeli and Lawrence (1994). This nanoparticle might interact with the skin in an adjuvant way. Figure 3 shows the potential nanoparticles and its action on the skin for topical applications. When there is a notorious damage to the skin surface, as it happens in skin disease and aged people, this might be a potential route for drug delivery, enhancing drug penetration. This may be observed for example, in patients diagnosed with ulcerated squamous cell carcinoma. Topical drug delivery can be improved by a better understanding of nanoparticle skin interaction and also by advanced particle engineering (Erdogan 2009). When dealing with topical drug delivery, it is very important to take into consideration the toxicity that might happen to the skin, so for this reason some in vitro and in vivo tests would be necessary in order to ascertain that these drugs will not provoke keratinocyte apoptosis through delivery, which is related to the difference between the shorter or longer phosphatidylcholine chains.

The Scientific Committee on Consumer Products (SCCP) stated that there are pieces of evidence that dealing with very small nanoparticles sized 10 nm or less, it is possible to observe the skin penetration into viable tissue. However, when dealing with nanoparticles larger than 20 nm in diameter, they can penetrate deeply into the hair follicle, but not reach viable tissue (SEC 2008).

#### Microemulsions for topical delivery

**Fig. 4:**
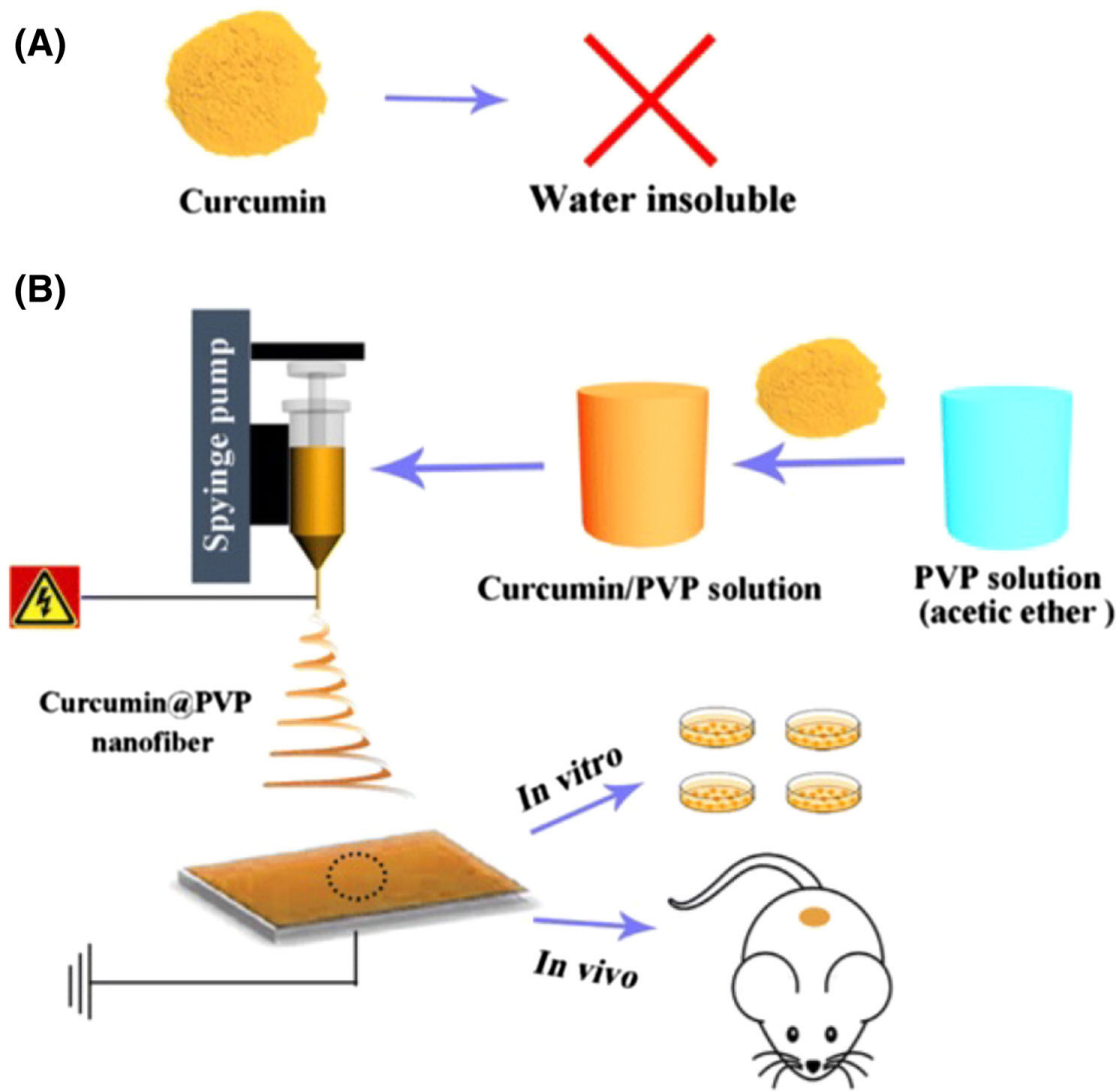
Case of study curcumin nanofibers, emulsion technique for spinning drug-loaded nanofibers for anticancer therapy. **a** Representative image of Curcumin polymer which is not soluble in water. **b** Representative Image of electrospinning process of CUR/ PVP nanofibers, and investigation of pharmacological effects in vitro and in vivo [Open access: (Wang et al. 2015)]

Hoar and Schulman (Kreilgaard 2002) first developed the theoretical microscopic emulsion by structural mixtures of cationic soap, alcohol, the oil phase, and water (Trotta et al. 1994). The microemulsion is described as a system of oil, water, and surfactants, which is a thermodynamic stable liquid solution, transparent, and single optically isotropic (Danielsson and Lindman 1981). It can be applied in a wide range of oil–water–surfactant composition, depending on the properties of the components involved in its formulation composition (Attwood et al. 1992). When designing a new microemulsion for pharmaceutical application, it is necessary to have a balance between the compounds forming the microencapsulation, in order to ascertain the non-toxic effects of agents involved, their high solubility, fulfilling the requirements for a better topical drug delivery with a high thermodynamic activity of the drug (Fig. 4) in the idea proposed to develop drug-loaded nanoparticles (Aboofazeli and Lawrence, 1994; Aboofazeli et al.^1995^).

### Limitation in nanoparticles and nanofibers applications

Novel research is required for better understanding of the mechanisms of nanofibers on the cellular signaling and biochemical pathways. It is also required to elucidate the genotype, the cell morphology, differentiation, growth, and proliferation (Ramakrishna et al. 2006). When dealing with characterization and design of these materials, it is important to take into consideration the size and shape, as this last characteristic may interfere with intracellular uptake and nanoparticle transportation activity (Caldorera-Moore et al. 2010). A desirable design of stable material is needed, in order to avoid harmful degradation materials. Potential use of new nanotechnologies regarding topical drug delivery, it is important to exploit the pH gradient of the skin, which might be either helpful or harmful regarding the degradation of the products (Hanson et al. 2002). The architecture of polymeric materials, once controlled, may improve the drug carrying capacity, stability, and solubility. In acidic pH environment, the drug is delivered into the cytoplasmic compartment of the cells and in this environment the polymer backbone is detached from the drug and it therefore destabilizes the endosomal membrane for the controlled delivery of the drugs.

Targeted drug delivery to melanoma with the aid of nanocarriers allows therapeutic drugs to accumulate in tumor tissues with a high concentration, facilitates the uptake and internalization of the drug-loaded NPs by tumor cells, and avoids off-target distribution which may lead to severe adverse effects. In addition, the incorporation of therapeutic agents such as chemotherapeutic drugs, metals or magnetic particles, proteins, nucleic acids, and vaccines, creates an ideal delivery system for the clinical treatment for metastatic melanoma. The development of products for medical application should be studied in vivo, because the chemicals might be toxic. At the moment, most of the nanofibers used are synthetic, but we should take into consideration the utilization of natural biopolymers. Although no actively targeted NPs are commercially available right now, the promising result obtained in the phase I/II clinical trials of these targeted NPs will offer the opportunity to carry out further pre-clinical and clinical investigations in melanoma targeting. At a cellular level, a variety of mechanism contributes to drug resistance. The p-glycoprotein (P-gp), is an example of a protein found in the membrane provoking drug efflux, solving this problem is possible, thus developing effective methods for drug delivery that could bypass or overcome the cellular resistance mechanisms in melanoma cancer therapy (Krishna and Mayer 2000; Vasir and Labhasetwar 2005).

## Case study

### Magnetic-based core–shell particles (MBCSPs)

**Fig. 5:**
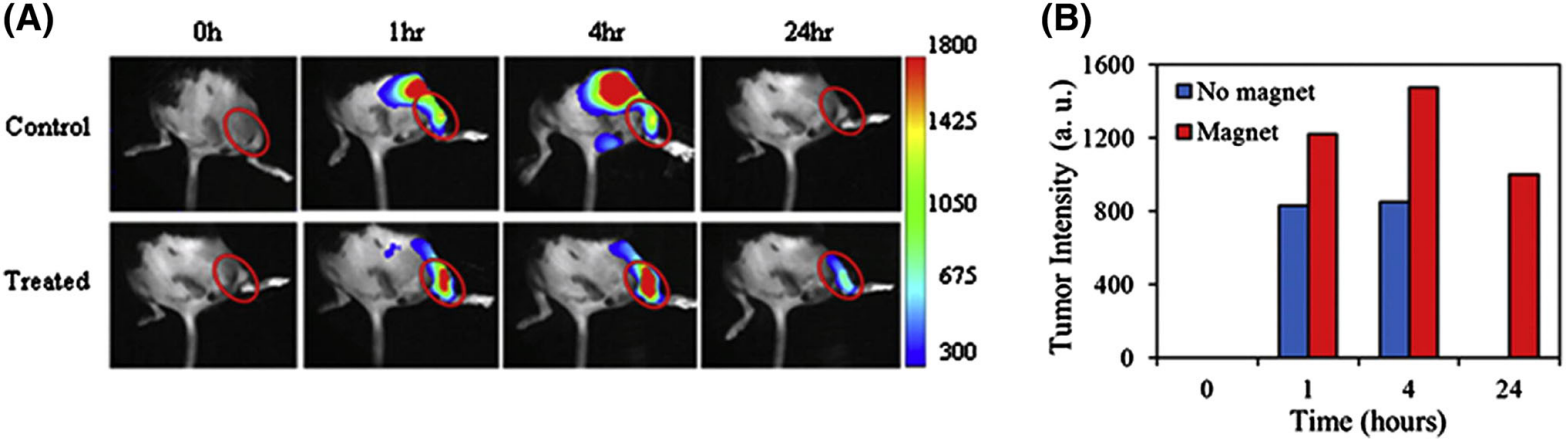
Magnetic-based core–shell particles for controlled release of anticancer drugs for melanoma cells. **a** Animals with no magnetic targeting, the signal of particles was detected intensively at earlier stages, rarely detected by 24 h after particle administration. On the contrary, for treated animals, a magnet was placed on *top* of tumor melanoma bearing, indicated with *red circle*. **b** Quantification of the fluorescence intensity, where it is possible to observe that the animals were treated, having the particle accumulation at the tumor in the presence of magnetic field [With permission from Elsevier (Wadajkar et al. 2012)

Wadajkar and co-workers reported the development of magnetic-based core–shell particles (MBCSPs) to target melanoma skin cancer cells B16F10 (Wadajkar et al. 2012). The MBCSPs, consist of thermoresponsive core of poly(lactic-*co*-glycolic acid) (PLGA) and shell of poly(*N*-isopropylacrylamide-acrylamide-allylamine) embedded with magnetite nanoparticles. Melanoma cancer cells were targeted by the conjugation of MBCSPs with Gly-Arg-Gly-Asp-Ser (GRGDS) peptides that specifically bind the receptors of melanoma cells *α*_*5*_ *β*_*3*_. The in vivo tumor targeting and biodistrubution were carried on orthotopic mouse model using B16F10 melanoma cell line to assess the magnetic targeting of the GRGDS–MBCSPs to tumors. The particles were loaded with NIR-797, for in vivo imaging as a fluorescent dye, as seen in Fig. 5. The induction of subcutaneous tumor implantation was done in the right flank of mice by the injection of 2 × 10^6^ cells suspended in 0.1 ml of PBS. The tumor size was monitored weekly, till it reached the size of 300 mm^3^. The animals were anesthetized by inhalation of 1% isoflurane. Finally, the suspension (100 µl of 5 mg/ml) of GRGDS–MBCSPs were injected intravenously, accordingly to the protocol described (Ma et al. 2007). The in vivo monitoring of the animals was performed at 0, 1, 4, and 24 h after particle administration. GRGDs-conjugated PLGA nanoparticles were observed to target tumors significantly once compared to non-conjugated nanoparticles (Danhier et al. 2009). In Fig. 5 it is possible to observe that the researchers could develop MBCSPs providing anticancer drug release via two mechanisms: one dependent on degradation, and the other dependent on temperature. The aggregation and the toxicity issues are reduced due to the encapsulation of magnetite in a biodegradable polymeric core, representing a sustained long-term drug release. The particles also exhibit efficient uptake by target melanoma cells and good cytocompatibility to healthy cells.

### Electrospun mats

Wang and co-workers (Wang et al. 2015), presented a study of electrospun mats with enhanced anticancer activity and bioavailability, in which they prepared electrospun mats based on curcumin (CUR)—loaded with polyvinyl pyrrolidone (PVP). Curcumin was used due to its properties such as: anticancer activity, anti-inflammatory, pharmacological activities, and antarthritic reported (Guo et al. 2011). Moreover, CUR has shown no toxicity in animals and humans, and no abnormality even when used at very high doses (Balaji and Chempakam 2010).

**Fig. 6:**
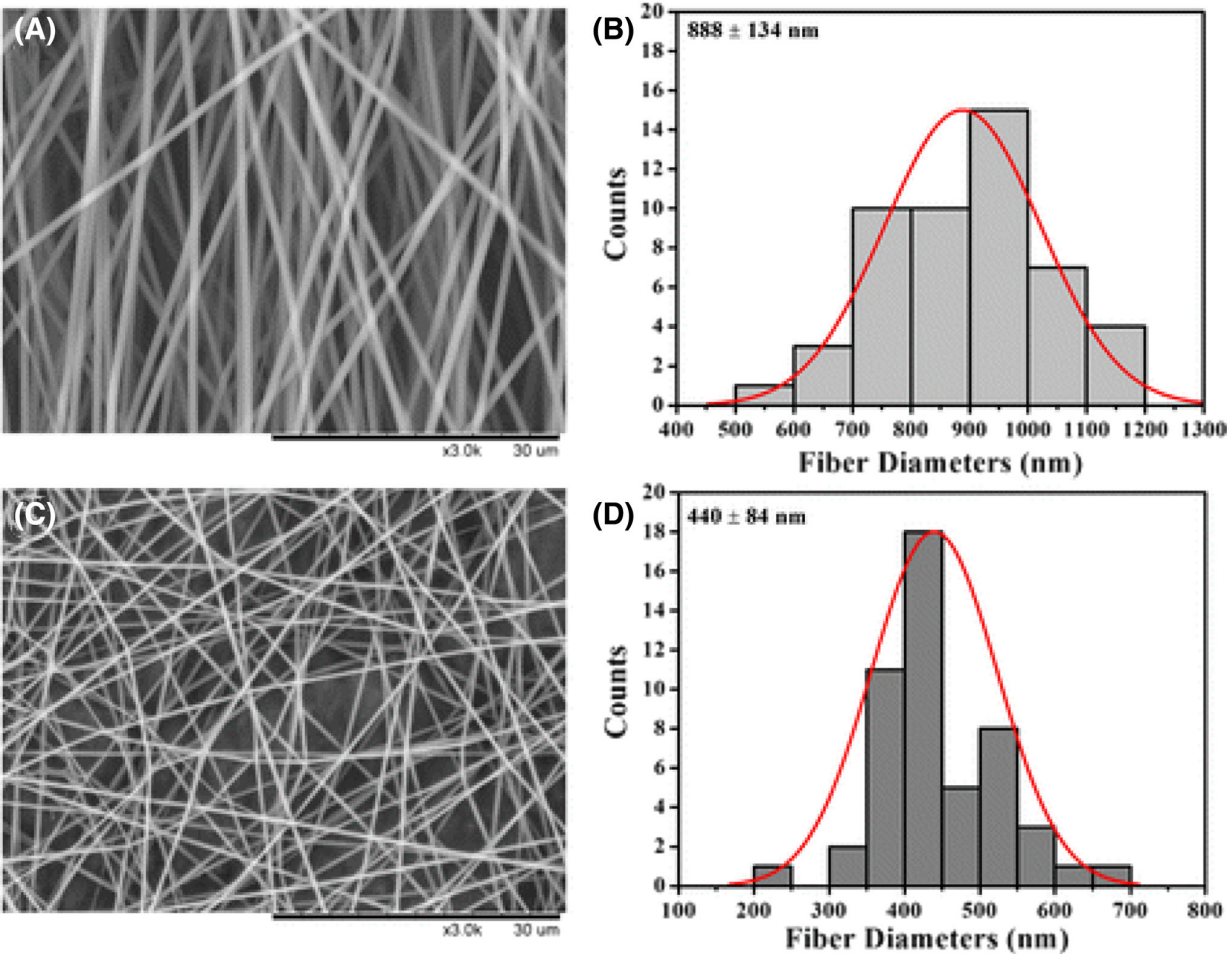
SEM images and distribution histogram. **a**, **b** Polyvinyl pyrrolidone (PVP) mats, **c**, **d** curcumin loaded with polyvinyl pyrrolidone (CUR/PVP) [Open Access: (Wang et al. 2015)]

The morphological structure of PVP and CUR/PVP is shown in Fig. 6. It can be observed that the mats are uniform and bead free. In Fig. 6a, b, the diameter of PVP fibers was 888 ± 134 nm. The CUR/PVP, Fig. 6c, d, diameter was decreased to 485 ± 123 nm, suggesting that the incorporation of CUR into PVP solution increased the electrospinning conductivity, enhanced the electrical drawing effect on the jet fluid, therefore, it decreased the fiber diameter (Zong et al. 2002).

**Fig. 7:**
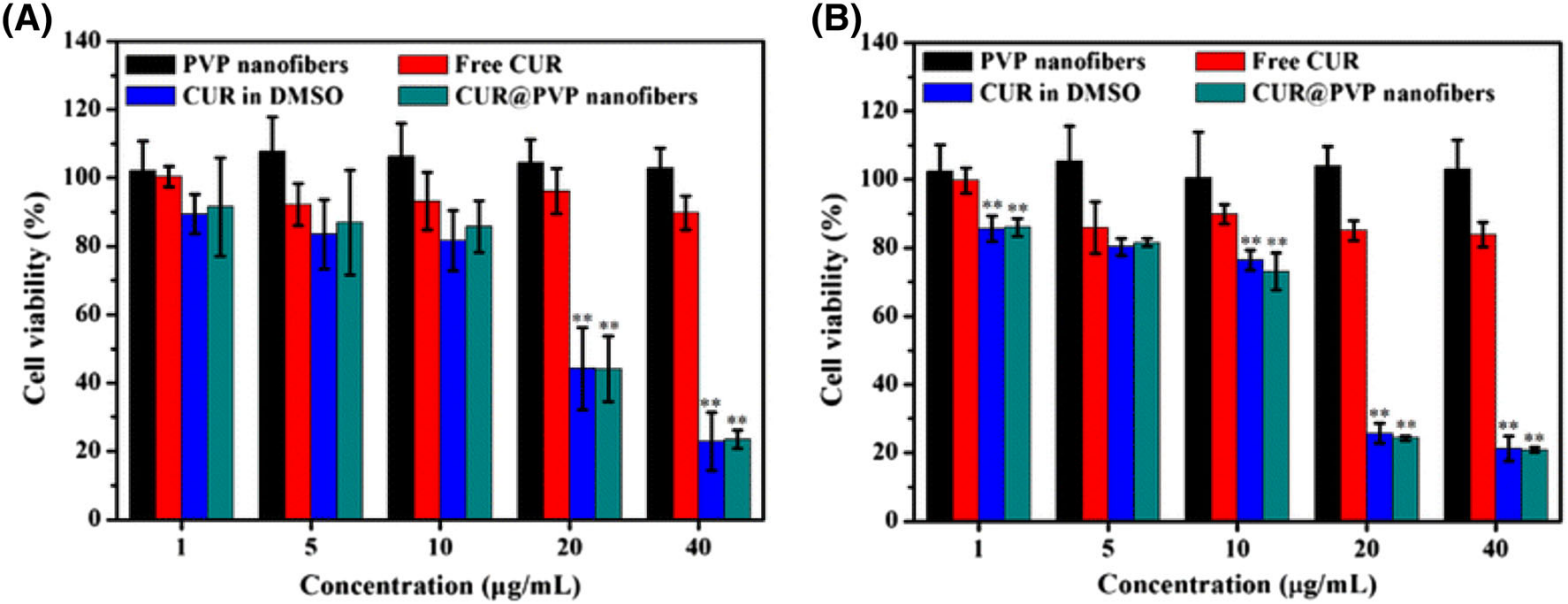
In vitro cell viability test of B16 cells. PVP, free CUR, Cur in DMSO, and CUR/PVP, in different concentrations from 1 to 40 µl/ml. The ** denotes significant difference compared to free CUR (*P* < 0.01) [Open access: (Wang et al. 2015)]

The cytotoxicity for murine melanoma cell lines, denoted as B16, was done on all electrospun mats. In the in vitro assay, the researchers seeded 5000 cells on 96-well plates and left it overnight. For the experiment, free CUR, CUR dissolved in DMSO, and CUR/PVP mats with different concentrations of curcumin in medium were added to the plates; these concentrations corresponded to 5, 10, 20, or 40 µg/ml CUR. As control, the cells were treated with PVP. After incubation for 24 and 72 h, in Fig. 7a and b, the wells were washed with PBS, added 90 µl of serum-free RPMI medium and 10 µl of cell counting kit-8 to each well. The plates were left into the incubator at 37° for 4 h. The readings determining the cell viability were taken by measuring the optical absorbance at 450 nm. As shown in Fig. 7, PVP mats show no cytotoxicity to B16. The drug concentration was related to the toxicity effect on B16 cells, as we can observe for concentrations over 10 µl, both CUR in DMSO and CUR/PVP decreased the cell viability for 24 and 72 h. The results suggest that CUR/PVP mats exhibit efficient cell proliferation effect on B16, compared to free CUR.

The in vivo pharmacokinetics (PK) test was humanely conducted in compliance with the Institutional Animal Care and Use Committee (IACUC) guidelines. In the test 48 male C57BL/6 mice were used. The melanoma cells B16 were administrated as subcutaneous allografts. The mice were shaved on their armpits and then a single injection of 1 × 10^6^ B16 cells in PBS was administrated. The CUR and CUR/PVP mats were administrated orally at a dosage of 25 mg/kg. The size of each tumor was measured everyday by the maximum diameter with a caliper. All the mice were euthanized by pentobarbital sodium overdose injection. Finally, the tumor of each mouse was carefully dissected from subcutaneous tissue and immersed into 4% formalin PBS solution for 48 h fixation.

**Fig. 8:**
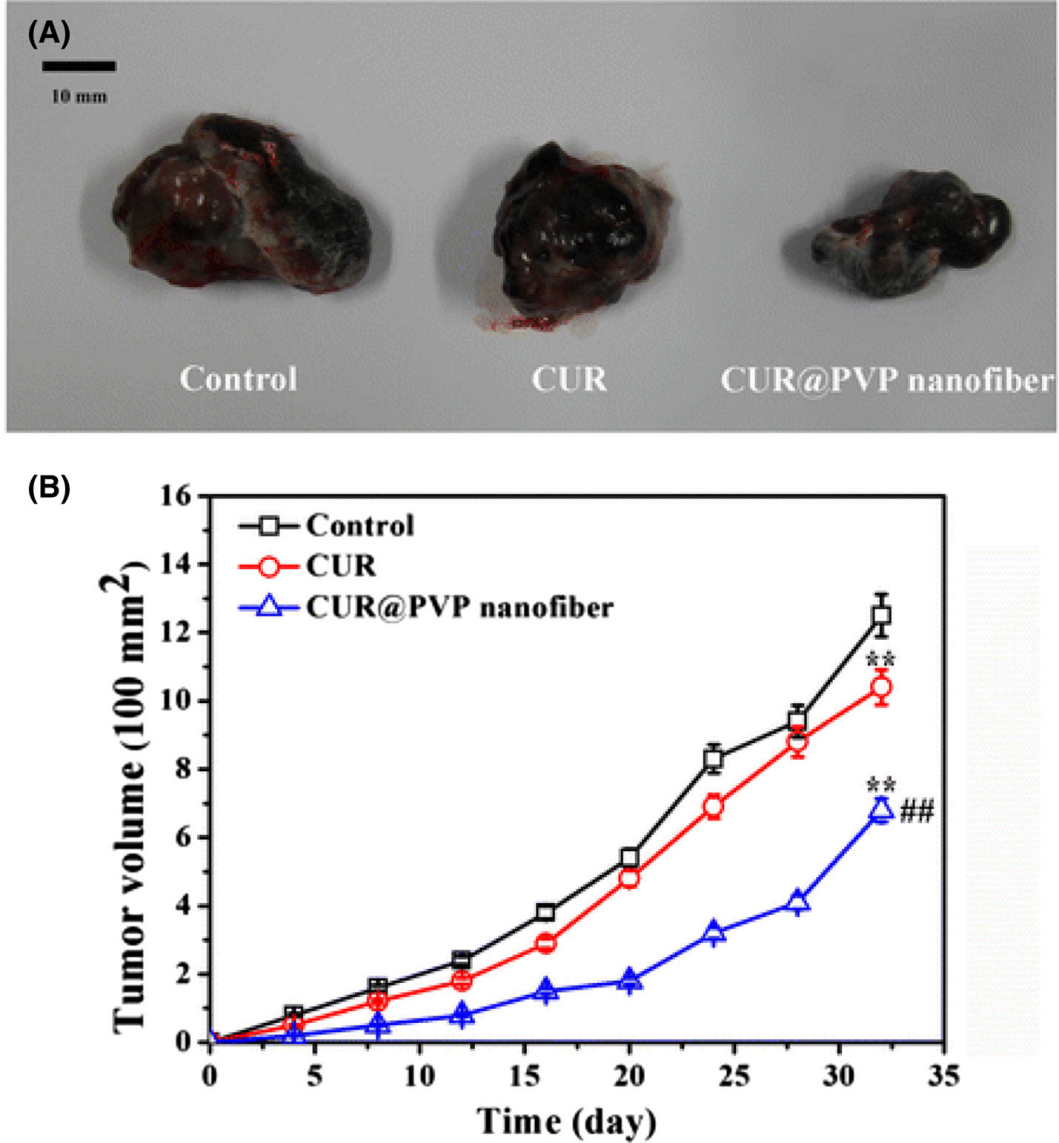
**a** Photo of the tumor at day 32; **b** tumor volume *x* time ^##^ denotes significant difference (*P* < 0.01) against CUR group and ** denotes significant difference (*P* < 0.01) against control group [Open access: (Wang et al. 2015)]

The analysis show that all animals have developed small nodules after 4 days. The tumor growth curve is presented in Fig. 8b. In animals which were treated as control PVP, the tumor was visibly faster than CUR/PVP group, and slightly quicker for those in pure CUR group (Fig. 8a). The group treated with CUR/PVP as we can observe in Fig. 8b displays much higher tumor inhibition efficiency compared to PVP and CUR groups, which are related to the enhanced bioavailability of the CUR nanosolid dispersion.

## Conclusion

Melanoma skin cancer should be considered by the scientists, physicians, and research community to cure the disease using novel drug delivery applications. The above details pointed out the possibilities to enhance the topical drug delivery after massaging the skin, or pulling out the follicles may be an alternative route for the therapeutic absorption. Once dealing with skin cancer, it is very important to observe the Breslow’s tumor thickness, which might be life-threatening; the deeper the cancer cells are located the more metastatic cancers possibly exist. Regarding topical drug delivery, it is important to exploit the pH gradient of the skin, which might be either helpful or harmful regarding the degradation of the products. The p-glycoprotein (P-gp) is a concern that scientists have to take into consideration when developing new drugs, in order to avoid drug efflux. It is also important to use nanotechnology for the development of new electrospun fibers that might work as BRAF inhibitors for the treatment of melanoma. We suggest further research in these agents to establish the possibility to develop topical drug delivery system by microemulsion or scaffolds for topical application. Only in this way we can target the tumor and design either drug-loaded nanofibers or nanoparticles which may yield the greatest returns in clinical melanoma therapy.
